# Distribution and regulatory roles of oxidized 5-methylcytosines in DNA and RNA of the basidiomycete fungi *Laccaria bicolor* and *Coprinopsis cinerea*

**DOI:** 10.1098/rsob.210302

**Published:** 2022-03-02

**Authors:** Janina Ličytė, Kotryna Kvederavičiūtė, Audronė Rukšėnaitė, Eglė Godliauskaitė, Povilas Gibas, Vita Tomkutė, Gražina Petraitytė, Viktoras Masevičius, Saulius Klimašauskas, Edita Kriukienė

**Affiliations:** Department of Biological DNA Modification, Institute of Biotechnology, Life Sciences Center, Vilnius University, Saulėtekio av. 7, Vilnius 10257, Lithuania

**Keywords:** 5hmC, 5fC, 5caC, Tet, fungi, Top-seq

## Abstract

The formation of three oxidative DNA 5-methylcytosine (5mC) modifications (oxi-mCs)—5-hydroxymethylcytosine (5hmC), 5-formylcytosine (5fC) and 5-carboxylcytosine (5caC)—by the TET/JBP family of dioxygenases prompted intensive studies of their functional roles in mammalian cells. However, the functional interplay of these less abundant modified nucleotides in other eukaryotic lineages remains poorly understood. We carried out a systematic study of the content and distribution of oxi-mCs in the DNA and RNA of the basidiomycetes *Laccaria bicolor* and *Coprinopsis cinerea,* which are established models to study DNA methylation and developmental and symbiotic processes. Quantitative liquid chromatography–tandem mass spectrometry revealed persistent but uneven occurrences of 5hmC, 5fC and 5caC in the DNA and RNA of the two organisms, which could be upregulated by vitamin C. 5caC in RNA (5carC) was predominantly found in non-ribosomal RNA, which potentially includes non-coding, messenger and small RNA species. Genome-wide mapping of 5hmC and 5fC using the single CG analysis techniques hmTOP-seq and foTOP-seq pointed at involvement of oxi-mCs in the regulation of gene expression and silencing of transposable elements. The implicated diverse roles of 5mC and oxi-mCs in the two fungi highlight the epigenetic importance of the latter modifications, which are often neglected in standard whole-genome bisulfite analyses.

## Graphical abstract

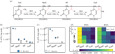

## 1. Introduction

**Figure 1.**
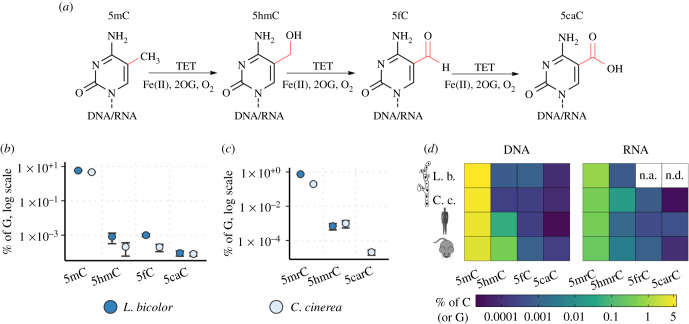
Oxidized 5-methylcytosine modifications (oxi-mCs). (*a*) Successive oxidation of 5mC performed by TET proteins. (*b*,*c*) Occurrence of cytosine modifications analysed by quantitative liquid chromatography–tandem mass spectrometry (HPLC-MS/MS) quantification in (*b*) DNA and (*c*) RNA of *Laccaria bicolor* and *Coprinopsis cinerea* grown under conventional conditions. Amounts of 5caC, 5fC, 5hmC and 5mC were calculated as the percentage of total G using a calibration curve of a corresponding nucleoside. At least two biological replicates were used. Oxidized 5mC derivatives were additionally purified by HPLC nucleoside fractionation prior to HPLC-MS/MS. A 5caC in RNA (5carC) signal was not detected in *L. bicolor* RNA. (*d*) Comparison of the abundance of modified cytosines in DNA and total RNA of the fungi *L. bicolor* and *C. cinerea* and mammals, presented as the percentage of total G (or C). Averaged approximate DNA data from various tissues were taken from [4] for mouse and from [5] for human; RNA data were taken from [6]. Data from untreated samples (without vitamin C or derivatization) were used for fungal DNA and RNA (5frC was not assessed in *L. bicolor* (n.a.) and 5carC was not detected (n.d.)). L. b., *L. bicolor*; C. c., *C. cinerea*.

5-Methylcytosine (5mC) is a widespread DNA modification found in organisms ranging from bacteria to mammals. In higher eukaryotes, it is an important epigenetic regulator that modulates gene expression and silencing of transposable elements (TEs) [1–3]. The much less abundant oxidized forms of 5mC are introduced through successive oxidation of 5mC to 5-hydroxymethylcytosine (5hmC), 5-formylcytosine (5fC) and 5-carboxylcytosine (5caC) by TET (ten–eleven translocation) oxygenases in the active DNA demethylation pathway (figure 1*a*), which is widely studied in mammals [5,7–11]. It is increasingly acknowledged that different oxidized 5mC modifications (oxi-mCs) have independent epigenetic roles besides being sole intermediates in the 5mC demethylation pathway; the most frequent of the oxi-mCs, 5hmC, has important roles in embryonic development and gene transcription regulation in healthy and cancerous tissues [12–14], while more rare 5fC and 5caC tend to accumulate at active enhancers [10,11,15–17], suggesting that they are implicated in gene activity regulation.

Besides widely explored 5mC oxidation in DNA by TET proteins *in vitro* and *in vivo*, these enzymes are also able to oxidize 5mC in RNA (5mrC) to all three oxi-mC forms (5hmrC, 5frC, 5carC) *in vitro* [18,19]. Furthermore, 5hmrC can be derived from 5mrC [20] *in vivo* with a contribution from TET dioxygenases [18], suggesting that 5mrC in RNA may undergo the same demethylation mechanism. 5mrC is widespread across both coding and non-coding RNAs from all domains of life [21]. Oxi-mCs were also found in the RNA of a variety of organisms: mammals, plants, insects [6,18,20,22,23]. Some studies provide evidence that they might affect RNA stability and translation, as demonstrated for 5hmrC in *Drosophila* [23]. 5carC has been recently discovered in mammalian RNA [6], but there is still little knowledge about its prevalence and functions compared with those for 5hmrC or 5frC [20,22,24,25].

TET enzymes belong to a large TET–J-binding protein (JBP) family. Sequence and phylogenetic analyses predicted origination of the TET-JPB family from bacteriophages, and usually a single or just a few copies of TET-JPB are found in animals, *Acanthamoeba*, certain algae, *Naegleria*, kinetoplastids, certain bacteria and phages. By contrast, fungi, especially basidiomycetes (mushrooms, rusts and smuts), display lineage-specific expansions with numerous *TET-JPB* genes, which are often associated with a unique class of TEs. The majority of TE-associated *TET-JPB* genes in basidiomycetes *Laccaria bicolor* and *Coprinopsis cinerea* (47 of 74 and 32 of 47 homologues, respectively) are predicted to be catalytically active [26–28].

Oxi-mCs have been found in *C. cinerea* DNA, where they were simultaneously mapped using single-molecule real-time (SMRT) sequencing and, additionally, by cytosine 5-methylenesulfonate immunoprecipitation (CMS-IP) for 5hmC [29]. However, the distribution of individual oxi-mCs in the genomes of *L. bicolor* and *C. cinerea* and the impact of individual oxi-mCs on gene expression and transposon regulation has not been explored in detail.

Both fungi belong to the order Agaricales, a large group of Basidiomycota. The relatively small genomes of *C. cinerea* and *L. bicolor* (approx. 37 and approx. 61 Mb, respectively) are sequenced and assembled (partially for *L. bicolor*) [30,31], and have been analysed in DNA methylation studies [3]. Saprotrophic *C. cinerea* is a classic fungal model organism that can be easily cultured under laboratory conditions, where it completes the life cycle in just two weeks and can be genetically manipulated [30,32]. *Laccaria bicolor* is an important ectomycorrhizal symbiont of hardwood and conifer species, which has been used to study the genes underlying the symbiosis interactions and ecological adaptation [31,33]. Because of the essential role of the fungi for tree growth and in the cycling of essential nutrients, ectomycorrhizal symbiosis is of global ecological and economic importance [34].

The biological functions of oxi-mCs in the DNA and RNA of phylogenetic groups of eukaryotes other than mammals are still underexplored. Here, we analysed the distribution of the 5mC oxidative forms in the nucleic acids of *L. bicolor* and *C. cinerea* by quantitative liquid chromatography–tandem mass spectrometry (HPLC-MS/MS) and whole-genome profiling, which demonstrated persistent occurrence of 5hmC and 5fC in their DNA and RNA and an almost exclusive presence of 5caC in long polyA depleted RNAs and small RNAs. High-resolution whole-genome mapping of 5hmC and 5fC highlighted their preferential distribution in TEs, which exert a strong gene regulatory role, and revealed the diverse influence of oxi-mCs and their predecessor 5mC on gene expression in the two fungi.

## 2. Material and methods

### 2.1. Cultivation of fungi

*Laccaria bicolor* (Maire) Orton S238N-H82 haploid monokaryotic strain (NCBI:txid486041) was kindly provided by Prof. Francis M. Martin. *Coprinopsis cinerea* haploid monokaryotic strain Okayama 7 (NCBI: txid240176) was obtained from the Fungal Genetics Stock Center (FGSC; no. 9003).

Mycelium of *C. cinerea* was maintained on solid (1% agar) yeast–malt–glucose (YMG) medium [35]. For nucleic acid extraction, 300 ml of liquid medium in a 1 l flask was inoculated with a small amount of mycelium and grown without shaking at 30°C for 14 days in the dark. For the enhancement of TET activity, mycelium was grown for 7 days and then treated with 5 mM vitamin C for 2 days (unless specified otherwise); untreated control was grown for the same time period.

*Laccaria bicolor* mycelium culture was maintained at 25°C in the dark on a solid (2% agar) modified Pachlewski medium (10 g l^−1^ glucose, 3 g l^−1^ malt extract, 0.25 g l^−1^ (NH_4_)_2_SO_4_, 0.5 g l^−1^ KH_2_PO_4_, 0.15 g l^−1^ MgSO_4_·7H_2_O, 0.05 g l^−1^ CaCl_2_, 0.025 g l^−1^ NaCl, 0.0012% FeCl_3_, 100 mg l^−1^ thiamine, 2.3 mg l^−1^ ZnSO_4_·7H_2_O, 8.5 mg l^−1^ H_3_BO_3_, 0.5 mg l^−1^ MnCl_2_·4H_2_O, 0.6 mg l^−1^ CuSO_4_·5H_2_O, 0.4 mg l^−1^ (NH_4_)_2_MoO_4_). For nucleic acid extraction, mycelium was collected after eight weeks from a liquid medium inoculated with a small amount of mycelium from agar plates. For vitamin C treatment, mycelium was incubated with 5 mM vitamin C for 2 days.

### 2.2. Purification of fungal DNA

Mycelium was crushed to a powder with a pinch of polyvinylpyrrolidone (PVP, K25) using a mortar, pestle and liquid nitrogen. Powder (approx. 1 ml) was incubated at 55°C for 90 min in 4.5 ml of lysis buffer (100 mM Tris–HCl pH 8.0, 20 mM EDTA, 2 M NaCl, 2% hexadecyltrimethylammonium bromide (CTAB), 2% PVP) with Proteinase K (Thermo Scientific). Then, DNA was extracted using chloroform and precipitated for 30 min at 4°C using 1.5 volumes of isopropanol and 0.4 volumes of 3 M sodium acetate pH 5.2.

DNA was dissolved in 10 mM Tris–HCl and treated with RNase A/T1 (Thermo Scientific) for 1.5 h at 37°C and then purified by chloroform extraction and ethanol precipitation. DNA quality was checked by agarose electrophoresis and the concentration was determined with a Qubit dsDNA HS Assay Kit (Invitrogen).

### 2.3. Fungal RNA purification

Mycelium was first crushed as described for DNA extraction. *C. cinerea* RNA for western dot-blot and *L. bicolor* RNA was extracted using RNAzol RT Reagent (MRC) according to the recommendations of the vendor.

For other experiments, total RNA of *C. cinerea* was extracted by a modified CTAB method [36]. Mycelium powder (approx. 100–500 µl) was incubated at 65°C for 10 min in 1 ml of lysis buffer (2% CTAB, 2% PVP K25, 100 mM Tris–HCl pH 8.0, 25 mM EDTA pH 8, 2 M NaCl) at 550 r.p.m. Then RNA was purified by chloroform extraction and ethanol precipitation with NaOAc. RNA quality was checked by agarose electrophoresis and the concentration was determined with a Qubit RNA HS Assay Kit (Invitrogen).

### 2.4. Control RNA preparation

Control RNA was synthesized by *in vitro* transcription using a TranscriptAid T7 High Yield Transcription kit (Thermo Scientific) as recommended by the vendor. A linearized pUC18-based plasmid carrying a recombinant sR47 (*Pyrococcus abyssi*) gene [37] was used as a DNA template. For synthesis of 5carC containing RNA, 25% of 5caCTP (TriLink BioTechnologies) from total cytidine triphosphate (CTP) was used in the reaction. RNAs were purified with an RNA Clean and Concentrator-5 kit (RCC; Zymo Research). 5carC amounts in the control RNA were evaluated using HPLC-MS/MS.

### 2.5. Analysis of 5caC by western dot–blot

Unmodified and 5carC-containing sR47 RNA was used as a control. For a positive DNA control, an oligonucleotide containing 5caC was used (5′-TAATAATAAA5caCCGTAATAATAATAAT; Metabion) and mixed to final amounts in nanograms as in samples with *dam– dcm–* lambda phage DNA (Thermo Scientific), which was also used as a negative control. RNA was denatured at 70°C for 10 min; DNA at 95°C for 10 min in water. Samples were loaded onto a Hybond-NX membrane (GE Healthcare Life Sciences, Amersham, UK), dried and cross-linked with UV for 3 min. Membrane was dyed with 0.04% methylene blue (Roth) in 0.3 M sodium acetate (pH 5.2) for 5 min and rinsed in water and washed for 5 min in phosphate-buffered saline (PBS) (137 mM NaCl, 2.7 mM KCl, 10 mM Na_2_HPO_4_, 1.8 mM KH_2_PO_4_) containing 0.1% Tween 20.

A membrane was blocked for 1 h at room temperature with 2% skimmed milk in Tris-buffered saline (TBS) (50 mM Tris-HCl pH 7.4, 150 mM NaCl). Then, it was incubated overnight at approximately 6°C with 1.5 µg of antibodies against 5caC (Active Motif catalogue no. 61225, RRID:AB_2793557) in 4 ml of TBS containing 2% milk and 0.1% Tween 20 and was washed three times for 5 min with TBS containing 0.1% Tween 20. Then, 1.6 µl of secondary goat horseradish peroxidase (HRP)-conjugated antibodies against rabbit (Active Motif catalogue no. 15015) was added in 4 ml buffer containing TBS, 2% milk and 0.1% Tween 20 and incubated for 1 h at room temperature. Washing was performed three times for 5 min with TBS containing 0.1% Tween 20 and two times with water, then the membrane was dyed with a chromogenic substrate, 3,3′,5,5′-tetramethylbenzidine (TMB; Sigma-Aldrich).

### 2.6. High-performance liquid chromatography coupled with tandem mass spectrometry

For quantification of DNA oxi-mCs, approximately 9–23 µg of DNA was digested and the nucleosides were fractionated and analysed with HPLC-MS/MS as in [15]. Unfractionated DNA was used for assessment of 5mC (20–50 ng) and, after vitamin C treatment (approx. 2 µg), for 5fC and 5hmC, which was analysed as in [15].

For analysis of oxi-mCs in RNA, approximately 20–24 µg *C. cinerea* RNA and 5 µg unmodified RNA containing standards for calibration was first fractionated into separate 5carC, 5hmrC and 5frC nucleoside fractions as for DNA in [15]. Unfractionated RNA was used for assessment of 5mrC (20–50 ng). Samples were analysed on an integrated high-performance liquid chromatography/electrospray ionization–tandem mass spectrometry (HPLC/ESI-MS/MS) system (Agilent 1290 Infinity/6410B triple quadrupole) equipped with a Supelco Discovery HS C18 column (7.5 cm × 2.1 mm, 3 µm) using a linear gradient of solvents at a flow of 0.3 ml min^−1^ at 30°C. Solvents A (0.0075% formic acid in water) and B (0.0075% formic acid in acetonitrile) were used with gradients as in [15] for each corresponding DNA modification. The mass spectrometer was operating in the positive ion MRM mode and the intensities of nucleoside-specific ion transitions were recorded: *m/z* rC 244.1 → 112, rG 284.1 → 152.1, 5mrC 258.1 → 126.1, 5carC 288.1 → 156, 5hmrC 274.1 → 142, frC 272.1 → 140. The ionization capillary voltage was 1800 V, the drying gas temperature was 150°C, the flow rate was 10 l min^−1^, the collision energy was 15 V and the cell accelerator was 7 V. Fragmentation was set at 80 V for 5fC and 100 V for other nucleosides.

For analysis of 5carC derivatization, the HPLC gradient was: 0–6 min, 0% B; 6–22 min, 0–100% B; 22–26 min, 100% B; 26–27 min, 100-0% B; 27–35 min, 0% B; or 0–6 min, 0% B; 6–13.5 min, 0–6% B; 13.5–21.5 min, 6–50% B; 22–26 min, 100% B; 26–27 min, 100-0% B; 27–35 min, 0% B. The ion transition for the 5carC derivative was recorded as *m/z* 432.2 → 300.1, the fragmentation was set at 80 V, the collision energy was 8 V and the cell accelerator was 5 V. 5carC containing synthetic RNA was derivatized along with the samples in each experiment to account for possible variations of reaction effectivity. Then, the derivatization efficiency and initial 5carC amounts were evaluated with MS/MS in this RNA compared with untreated 5carC RNA. This RNA was then mixed with 5 µg unmodified RNA and used for 5carC-derivative calibration. The dependency of the 5carC-derivative MS/MS signal area from the initial 5carC amount was calculated. Signals could be quantified when present within a linear range of the calibration curve and could only be detected when the signal-to-noise ratio was ≥3.

High-resolution mass spectra of the 5carC derivatization product were acquired on a Q-TOF 6250 mass spectrometer (Agilent) equipped with a dual-ESI source.

### 2.7. Synthesis of the (4-aminomethyl)benzylazide and its hydrochloride salt


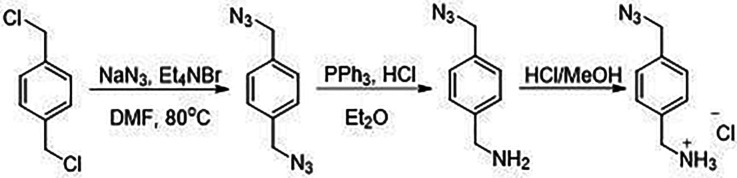


#### 2.7.1. 1,4-Bis(azidomethyl)benzene

To a stirred solution of *α*,*α*′-dichloro-*p*-xylene (4.0 g, 22.9 mmol) in *N*,*N*-dimethylformamide (DMF) (60 ml) sodium azide (8.17 g, 125.7 mmol) and tetrabutylammonium bromide (0.74 g, 2.29 mmol) were added. The reaction mixture was left to stir for 24 h at 80°C (sand bath). Then the solvent was evaporated under reduced pressure to dryness and water was added. The obtained solution was extracted with diethyl ether (3 × 50 ml), the organic layers were combined, dried over sodium sulfate and evaporated to give a colourless solid (4.15 g, 96%). ^1^H NMR (CDCl_3_, 400 MHz) *δ*: 7.42 (s, 4H, Ar*H*), 4.61 (s, 4H, 2 × C*H_2_*).

#### 2.7.2. 4-(Azidomethyl)benzylamine

Synthesis was performed according to [38]. To a mixture of 1,4-bis(azidomethyl)benzene (0.2 g, 1.06 mmol) in Et_2_O (6 ml) and HCl (4.5 ml, 1 M), PPh_3_ (0.36 g, 1.38 mmol) was added, and the heterogeneous mixture was stirred vigorously for 24 h. The organic layer was then separated and the aqueous layer was extracted with Et_2_O (3 × 5 ml) to remove the Ph_3_PO and unreacted starting materials. The pH of the aqueous layer was adjusted to 10 by adding KOH solution (2.5 M) and then extracted with CH_2_Cl_2_ (4 × 5 ml). The combined extracts were washed with brine, dried (Mg_2_SO_4_), filtered and evaporated under vacuum to give the product, which was a pale yellow oil (0.1 g, 58%). It was used in the next reaction without further purification. ^1^H NMR (CDCl_3_, 400 MHz) *δ*: 7.36 (d, *J* = 8.0 Hz, 2*H*, Ar*H*), 7.31 (d, *J* = 8.0 Hz, 2H, Ar*H*), 4.34 (s, 2H, C*H*_2_N_3_), 3.91 (s, 2H, C*H*_2_NH_2_), 1.56 (s, 2H, N*H_2_*).

#### 2.7.3. 4-(Azidomethyl)benzylamine hydrochloride

4-(Azidomethyl)benzylamine (0.1 g, 0.62 mmol) was stirred in 1.1 M hydrochloride methanolic solution at room temperature for 30 min, then the solution was evaporated. The resulting solid was washed with diethyl ether and dried to yield a yellowish solid (0.11 g, 90%). ^1^H NMR (DMSO-*d*_6_, 400 MHz) *δ*: 8.52 (br.s, 3H, N*H_3_*), 7.54 (d, *J* = 8.0 Hz, 2*H*, Ar*H*), 7.42 (d, *J* = 8.0 Hz, 2H, Ar*H*), 4.46 (s, 2H, C*H*_2_N_3_), 4.03 (s, 2H, C*H*_2_NH_3_).

### 2.8. Derivatization of 5carC-containing RNA

Derivatization of 5carC-RNA was based on [39]. Before the reaction, RNA was denatured at 70°C for 10 min in water and chilled on ice. Derivatization was done in two steps. For optimization of the reaction in the first step, 5carC containing sr47 RNA was incubated in 50 µl of 10 mM 2-(*N*-morpholino)ethanesulfonic acid (MES) buffer (pH 6), containing 20 mM *N*-hydroxysuccinimide (NHS; Sigma) with four different combinations of other reagents: (a) 2 mM EDC (1-ethyl-3-[3-dimethylaminopropyl]-carbodiimide hydrochloride; Sigma); (b) 8 mM EDC; (c) 2 mM EDC, 45% dimethyl sulfoxide (DMSO); (d) 8 mM EDC, 45% DMSO. NHS and EDC were freshly prepared, mixed together and added last to the reaction. The reaction in the first step was incubated for 30 min at 37°C, with shaking at 550 r.p.m. In the second step, in a final 150 µl volume 10 mM (4-aminomethyl)benzylazide hydrochloride, sodium phosphate (pH 7.5, 100 mM) and 150 mM NaCl were added and incubated for 1 h at 37°C, with shaking at 550 r.p.m. Reactions were diluted 2.5 times with water and RNAs then purified with an RNA Clean and Concentrator kit (Zymo Research).

For further derivatization of fungal RNAs (d) conditions were used in the first step; 0.7–25 µg RNA was incubated in a 60 µl volume in the first step and in the final 180 µl in the second step.

### 2.9. Fungal RNA purification into separate fractions

RNA was purified into large (longer than 200 nt) and small (shorter than 200 nt) fractions with a RNA Clean and Concentrator-5 kit (Zymo Research). PolyA RNA was purified from the large RNA fraction (100–106 µg) using a Dynabeads mRNA Purification Kit (Invitrogen). Large ribosomal RNAs were gel-purified from low melting point agarose using Agarase (Thermo Scientific) according to the recommendations of the vendor. Fractions of small RNAs were purified by polyacrylamide gel electrophoresis (PAGE): crushed gel was incubated in an elution buffer (0.5 M CH_3_COONH_4_, 0.1 mM EDTA, 0.1% SDS) for 2 h at 37°C with shaking at 600 r.p.m. Samples were filtered through a Ultrafree-MC Centrifugal Filter (Millipore) and RNA was precipitated using ethanol.

### 2.10. Evaluation of 5hmC and 5fC enrichment using control DNA fragments

The 188 bp model DNA fragments containing one 5hmCG or 5fCG site in one strand were produced from mouse gDNA by polymerase chain reaction (PCR) amplification as in [40]. The CG in the other strand was methylated using a wild-type SssI (Thermo Scientific) according to the vendor's protocol. Model DNA fragments were mixed with sonicated *L. bicolor* DNA at a ratio of 13 : 1287 ng to imitate the low abundance of modifications.

We used modified hMe-Seal and fC-Seal protocols [9,41] for detection of 5hmC and 5fC. For detection of 5fC, its reduction to 5hmC was performed with NaBH_4_ as in [15]. To discriminate 5fC from 5hmC in natural genomic DNA, 5hmC was protected by glycosylation. For 5hmC protection, glycosylation of the 5hmC/5mC fragment with unmodified UDP-glucose was performed as a control before labelling as described in [15]. 5hmC, reduced 5fC or protected 5hmC containing DNA fragments were labelled using 50 µM UDP-6-azide-glucose (Jena Bioscience) and T4 β-glucosyltransferase (T4 BGT; Thermo Scientific) using 10 U for 1 µg of DNA in a 50 µl reaction. The reaction was incubated for 2 h at 37°C, followed by enzyme inactivation at 65°C for 20 min and purification using a DNA Clean and Concentrator kit (Zymo Research).

Biotinylation was then performed by adding DBCO-S-S-PEG3-biotin (Jena Bioscience) to a 0.4 mM concentration in 10 mM Tris-HCl (pH 8.5) buffer, reactions were incubated at 37°C for 2 h and DNA was purified with a DNA Clean and Concentrator kit (Zymo Research). DNA was then enriched as follows: 0.1 mg of Dynabeads MyOne Streptavidin C1 (Thermo Scientific) was incubated with DNA in 10 mM Tris-HCl (pH 8.5), 1 M NaCl and 0.01% Tween 20 (Roth) at room temperature for 3 h on a roller. The beads were washed three times with 15 mM Tris-HCl (pH 7.5), 1.5 mM EDTA, 3 M NaCl, 0.01% Tween 20 and twice with 5 mM Tris-HCl (pH 7.5), 0.5 mM EDTA, 1 M NaCl and 0.01% of Tween 20.

The amounts of DNA in the bead and supernatant fractions were evaluated by qPCR using a Maxima SybrGreen qPCR Master Mix (Thermo Scientific); 0.3 µM of each primer pair was used in each reaction. The amplification programme was set as: 95°C for 10 min, 40 cycles at 95°C for 15 s, 60°C for 1 min on a Rotor-Gene Q real-time PCR system (Qiagen).

### 2.11. Preparation of hMe-seal and fC-seal libraries

Genomic DNA was sonicated on a Bioruptor UCD-200 (Diagenode) instrument in 10 mM Tris-HCl (pH 8.5) to on average 200–250 bp fragments and purified using a DNA Clean and Concentrator kit (Zymo Research).

5hmC protection and 5fC reduction to 5hmC was performed as in [15]. To achieve similar processing of 5fC and 5hmC samples, the same purification steps were performed with both samples, but omitting glycosylation with unmodified UDP-glucose and reduction with NaBH_4_ for 5hmC. An aliquot of 2.6–2.7 µg DNA was labelled using UDP-6-azide-glucose and T4 BGT (Thermo Scientific) as described above (see Evaluation of 5hmC and 5fC enrichment using control DNA samples). Two control DNA samples were prepared: for 5hmC control samples, BGT was omitted in the labelling step; for controlling 5hmC protection in 5fC samples, glucosylated DNA without the reduction step was used.

The biotinylation step was performed in a 130 µl reaction (as described in Evaluation of 5hmC and 5fC enrichment using control DNA samples), and then DNA was end-filled using a DNA End Repair Kit (Thermo Scientific) according to the vendor's recommendations and purified using a DNA Clean and Concentrator kit (Zymo Research). Biotinylated DNA was then enriched as described above (see Evaluation of 5hmC and 5fC enrichment using control DNA samples) in 40 μl of 10 mM Tris-HCl (pH 8.5), 1 M NaCl and 0.01% Tween 20 (Roth) using 0.1 mg of Dynabeads MyOne Streptavidin C1 (Thermo Scientific) and recovered by resuspending beads in 20 µl of 50 mM dithiothreitol, 60 mM Tris (pH 7.8), followed by a 1 h incubation on a roller. Released DNA was separated from the beads.

DNA was ligated overnight at 22°C to 4.2 pmol of Ion Torrent barcoded adapters (Kapa Biosystems) in a 30 µl reaction containing 0.5 mM ATP, 10 mM MgCl_2_, 5% PEG 4000 and 7.5 U of T4 DNA ligase (Thermo Scientific). Adapter removal was then performed using a MagJet NGS Cleanup and Size-selection kit (Thermo Scientific). Then, adaptor-ligated DNA was incubated with 10 mM 2-mercaptoethanol for 10 min at room temperature (to preclude inadvertent formation of inter-nucleotide disulfide cross-links), followed by PCR in 50 µl of Taq buffer with (NH_4_)_2_SO_4_ supplemented with 0.2 mM deoxynucleoside triphosphate (dNTP), 2 mM MgCl_2_, 1 μM primers and 2.5 U of Taq DNA polymerase (Thermo Scientific). PCR cycling conditions were: 1 min 50°C, 5 min 72°C, 4 min 94°C, 1 min 65°C, 5 min 72°C, 15 cycles of 1 min 94°C, 1 min 65°C, 1 min 72°C and the final extension step was at 72°C for 5 min. The libraries were size-selected for 300 bp fragments (MagJet NGS Cleanup and Size-selection kit, Thermo Scientific) and subjected to Ion Proton (Thermo Scientific) sequencing.

### 2.12. Preparation of hmTOP-seq and foTOP-seq libraries

DNA was sonicated to yield approximately 200–250 bp fragments with a Covaris M220 instrument and purified using a DNA Clean and Concentrator kit (Zymo Research). 5fC reduction to 5hmC, 5hmC protection and azide-labelling of 5hmC (5.8 µg of DNA in this step) were performed as described above for the hMe-Seal and fC-Seal libraries. Then, DNA was end-filled for 15 min at 20°C using a DNA End Repair Kit (Thermo Scientific) according to the vendor's recommendations and purified using a GeneJet Purification Kit (Thermo Scientific). DNA was then dA-tailed using 30 U of Klenow polymerase (Thermo Scientific) in 120 μl of its buffer (Thermo Scientific) in the presence of 0.5 mM dATP at 37°C for 45 min, enzyme inactivated at 75°C for 15 min followed by purification through GeneJet (Thermo Scientific) columns. Partially complementary annealed adaptors A1/A2 (2.25 μM, A1 5′ P-GATTGGAAGAGTGGTTCAGCAGGAATGCTGAG and A2 5′ ACACTCTTTCCCTACATGACACTCTTCCAATCT) were ligated to DNA using 30 U of T4 DNA ligase (Thermo Scientific) in 120 µl of its buffer (Thermo Scientific) at 22°C overnight, followed by thermal inactivation at 65°C for 10 min and column purification (DNA Clean and Concentrator; Zymo Research).

TOP-seq library enrichment and preparation were then done as in [15] and libraries were subjected to Ion Proton (Thermo Scientific) sequencing.

### 2.13. Preparation of RNA libraries

*Laccaria bicolor* RNA was extracted using RNAzol RT Reagent (MRC), treated with DNaseI (Thermo Scientific) as recommended by the vendor and the longer than 200 nt fraction was purified using RCC (Zymo Research). Then, ribosomal RNAs were depleted using a Ribo-Zero Gold rRNA Removal Kit (yeast; Illumina) according to recommendations of the vendor and the longer than 200 nt fraction was purified using RCC (Zymo Research). RNA libraries were further prepared and sequenced at Thermo Fisher Scientific Baltics, using a Collibri Stranded RNA Library Prep Kit (Invitrogen) for Illumina (approx. 200 M pair-end reads per sample).

### 2.14. Analysed sequences and sequence annotations

Whole-genome sequences and annotations were downloaded from the Joint Genome Institute Genome portal ([42]; https://genome.jgi.doe.gov/portal/; RRID:SCR_002383): the V2 version was used for *L. bicolor* [31] and V1 for *C. cinerea* [30]. Bisulfite sequencing data were downloaded from NCBI SRA PRJNA122153 [3]. *Coprinopsis cinerea* polyA RNA-seq data were downloaded from NCBI SRA—PRJEB4912 [43].

Protein domains were identified using InterProScan v. 86.0 ([44]; https://github.com/ebi-pf-team/interproscanhttp://www.ebi.ac.uk/Tools/pfa/iprscan/; RRID:SCR_005829) using standard parameters and look-up of corresponding Gene Ontology (GO) terms and Pathway annotations (–goterms and -pa options). RepeatModeler V2.0.1 (http://www.repeatmasker.org/RepeatModeler/; RRID:SCR_015027) and RepeatMasker V4.1.0 (https://www.repeatmasker.org/, RRID:SCR_012954) were used to identify repeats in each genome (standard parameters were used). TE classes were assigned using Censor [45] at the Genetic Information Research Institute (GIRI) (https://www.girinst.org/; RRID:SCR_012762). TE clusters were defined as regions rich in TEs and were identified using an in-house script. In brief, the genome was divided into non-overlapping windows of 100 bp length and windows with at least 25% TE content were identified. Identified windows were merged into core clusters (distance between windows ≤ 100 bp), and core clusters within less than a 10 kb distance were further merged. We then selected clusters that were at least 5 kb in length and the TE content was at least 50%. Final TE clusters were then made by extending identified clusters by 5 kb on both sides.

Unique genes were identified using an in-house script. In brief, local blastp ([46]; https://ftp.ncbi.nlm.nih.gov/blast/executables/blast+/LATEST/; RRID:SCR_001010) was used to identify similar protein sequences. Sequences with a similarity threshold of at least 95% and pairwise alignment coverage of at least 90% (up to 5% unmatched regions at both the N and C ends) were identified as non-unique sequences. TET genes were identified as those belonging to the TET-JPB superfamily (cl24253) using an online NCBI conserved domain database search (http://www.ncbi.nlm.nih.gov/cdd; RRID:SCR_002077).

### 2.15. Analysis of DNA sequencing data

hMe-Seal and fC-Seal data were analysed using a custom analysis workflow. In brief, raw reads were filtered by length (minimum length 15) and trimmed (PHRED > 20) using the FASTX-Toolkit (http://hannonlab.cshl.edu/fastx_toolkit/; RRID:SCR_005534), mapped using BWA ([47]; http://bio-bwa.sourceforge.net/; RRID:SCR_010910) and filtered to contain only MAPQ ≥ 30 reads. PCR duplicates were removed based on most left and most right mapping positions using a custom R script. Deduplicated data were downsampled to equal size per modification and organism. The reference genome was then divided into non-overlapping 200 bp windows and coverage per window was calculated. To remove background signals, we evaluated each window in a sample and its control: if sample coverage was at least 1.2 times higher than control sample coverage, the difference in coverage was used as a modification level within a window.

WGBS sequencing data were analysed using the BISMARK ([48]; http://www.bioinformatics.babraham.ac.uk/projects/bismark/; RRID:SCR_005604) pipeline with default settings. For the CG fraction analysis, only CGs covered with at least five reads were selected for further analysis. For enrichment analysis, CGs methylated more than 50% were used.

Raw hmTOP-seq/foTOP-seq data were analysed as described in [49] with several modifications: only reads longer than 30 bp were selected for mapping, which was performed with hisat2 ([50]; https://daehwankimlab.github.io/hisat2/; RRID:SCR_015530; –no-spliced-alignment was used to prevent spliced alignments), and only those CGs that showed coverage of at least two in the sample of combined technical replicates and scaffolds/chromosomes longer than 100 kb were used for the analysis.

### 2.16. Analysis of RNA sequencing

The quality of the raw data was evaluated using the FASTQC program (https://www.bioinformatics.babraham.ac.uk/projects/fastqc/; RRID:SCR_014583) and reads were trimmed using BBDUK from BBMAP packages (sourceforge.net/projects/bbmap/; RRID:SCR_016965) using the manufacturer's recommendations. Data were mapped using the hisat2 [50] program (https://daehwankimlab.github.io/hisat2/; RRID:SCR_015530; used options –no-unal –no-mixed –no-discordant –dta) using *L. bicolor* V2 [31] and *C. cinerea* [30] reference genomes and deduplicated using samtools markdup v. 1.12 ([51]; http://www.htslib.org; RRID:SCR_002105). StringTie v. 2.0.3 ([52]; https://ccb.jhu.edu/software/stringtie/; RRID:SCR_016323) was used to evaluate gene expression in TPM (transcripts per million) and DESeq2 ([53]; https://bioconductor.org/packages/release/bioc/html/DESeq2.html; RRID:SCR_015687) was used to identify differentially expressed genes. Expressed genes were identified as TPM >0.05.

### 2.17. Statistical analysis

The modification fraction was calculated using a formula *N*_modCG_/*N*_totalCG_, where *N*_modCG_ was the number of modified CGs and *N*_totalCG_ was the total number of CGs per gene. Expression and modification groups (deciles and quartiles) were calculated using only non-zero expression/modification genes. Low-/high-modification genes were defined as genes falling within the first or last decile. Enrichment analysis was performed by computing the contingency table for all or selected CGs (or 200-bp non-overlapping bins for hMe-Seal and fC-Seal) overlapping with a genomic region. Fisher's exact test was used to estimate the odds ratio and *p-*value. All statistical calculations were performed using R 3.4.0.3 (https://www.r-project.org; RRID:SCR_001905).

## 3. Results

### 3.1. Detection of oxidative forms of 5mC in *L. bicolor* and *C. cinerea*

We measured the global amounts of oxi-mCs in DNA extracted from the mycelium of *L. bicolor* and *C. cinerea* by western dot–blot and HPLC-MS/MS analysis. By HPLC-MS/MS, we observed four to five times higher amounts of 5hmC and 5fC in *L. bicolor* (approx. 0.0008 and approx. 0.001% of G) than in *C. cinerea* (approx. 0.0002%). In each of the two fungi, the amounts of 5fC and 5hmC were similar (figure 1*b,d*), in contrast to mammalian DNA, in which 5hmC is the most abundant of oxi-mCs (for example, approximately 0.06% 5hmC, approximately 0.0007% 5fC, approximately 0.0002% 5caC of C in mouse embryonic stem cells; [4]), while levels of 5caC were the lowest (approx. 0.0001%) of all oxi-mCs (see also figure 1*d*). These results contrasted with the western dot–blot analyses using the antibodies raised against 5caC (electronic supplementary material, figure S1A), which showed a high 5caC signal in *C. cinerea* DNA (electronic supplementary material, figure S1A). Therefore, we hypothesized that the 5caC signal observed in DNA by western dot–blot assays might appear to be due to cross-reactivity of the 5caC antibodies with some impurities derived from fungal extracts, or the presence of residual amounts of RNA which might also contain 5caC. Indeed, we detected 5caC in *C. cinerea* RNA by western dot–blot (electronic supplementary material, figure S1A) and confirmed the presence of 5carC in RNA by HPLC-MS/MS (figure 1*c*; electronic supplementary material, figure S1B), although its amounts were much lower than could be expected from the western dot–blot analysis (electronic supplementary material, figure S1A), thereby indicating a possible cross-reactivity with unknown components of fungal extracts. Importantly, the observed 5mrC, 5hmrC (figure 1*c*) and 5frC (electronic supplementary material, figure S1B) levels were similar to those reported in mammalian RNA (percentage of G: 5mrC approx. 0.25%; 5hmrC approximately 0.004%; 5carC approximately 0.0001% [6]; see also [20]; figure 1*d*).

### 3.2. Distribution of 5caC in RNA

We sought to explore the abundance and distribution of 5carC in RNA of both fungi by HPLC-MS/MS. The quantification of 5carC is a laborious task because, first, it requires high amounts of RNA (up to approx. 20 ug) and, second, in order to separate 5carC from the abundant cytosine that has a similar retention time in MS/MS, HPLC nucleoside fractionation and purification have to be performed prior to MS. To enhance the detection of 5carC, we employed a method used for chemical derivatization of 5caC in DNA [17,39], in which (4-aminomethyl)benzylazide is conjugated to the carboxyl group through the EDC chemistry. Under our optimized conditions (see electronic supplementary material, Methods and figure S2A), approximately 61% of 5carC was derivatized in synthetic RNA, resembling the reactivity of 5caC in DNA [39]. Of note, the MS signal intensity of the 5carC derivative increased approximately 20 times as compared with underivatized 5carC (electronic supplementary material, figure S2B) and its different retention time eliminated the need for prior fractionation. Moreover, the MS signal of the 5carC derivative showed a linear dependence on 5carC amounts in input samples (electronic supplementary material, figure S2D) and high selectivity, as the signal was well detected in the samples mixed with different amounts of unmodified RNA (electronic supplementary material, figure S2E). Since, on average, as little as 0.6 fmol of derivatized 5carC could be detected, the developed derivatization approach is well suited for measurements of 5carC in fungi and other biological samples.

**Figure 2.**
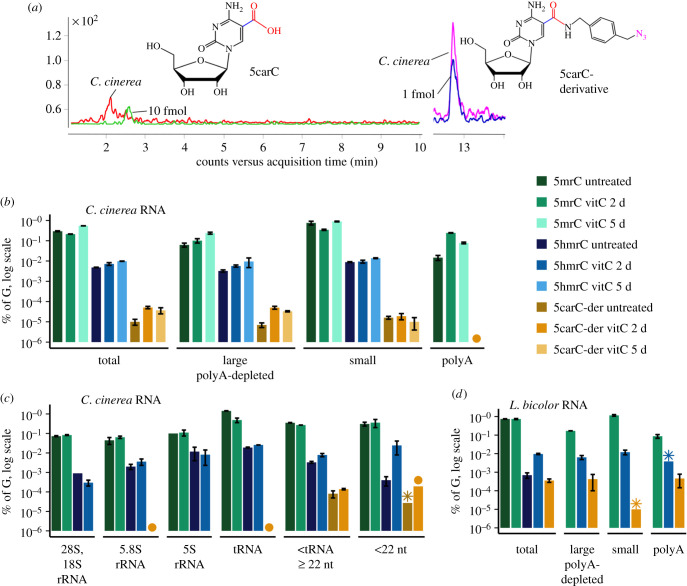
HPLC-MS/MS quantification of 5carC using chemical derivatization in RNA of basidiomycete fungi *L. bicolor* and *C. cinerea*. (*a*) Detection of 5carC using chemical derivatization with (4-aminomethyl)benzylazide. MS/MS signal of 5carC (red, 20 µg RNA) and 5carC derivative (magenta, 5 µg RNA) in *C. cinerea* RNA. A 5carC standard is shown in green (10 fmol), a 5carC derivative in blue (1 fmol). (*b*,*c*) HPLC-MS/MS analysis of the chemically derivatized 5carC in different RNA fractions of *C. cinerea* and (*d*) *L. bicolor*. Fungi were treated with vitamin C (vitC) for 2 (*b*,*c*,*d*) or 5 days (*b*). Untreated *L. bicolor* RNA was not fractionated, as it did not show a 5carC signal. Two biological replicates were used. Stars denote the samples with the detected and quantified signal (within a linear range of the calibration curve) in only one of the two replicates; circles denote where the signal was detectable but could not be quantified (was outside a linear range of the calibration curve) in one of the two replicates; samples which did not show any signal are not included (see also electronic supplementary material, table S1). Total, total RNA; small, shorter than 200 nt RNA fraction enriched by column purification; large polyA-depleted, longer than 200 nt enriched RNAs, from which polyA tail-containing RNAs (polyA) were depleted; <tRNA ≥22 nt, RNAs shorter than tRNA and longer than 22 nt; <22 nt, RNAs shorter than 22 nt. The small (shorter than 200 nt RNA) fraction includes 5.8S rRNA, 5S rRNA, tRNA and RNAs shorter than tRNA; large polyA-depleted RNA fraction includes 28S, 18S RNAs and other longer than 200 nt RNAs (see also electronic supplementary material, figure S3B).

Under standard cultivation conditions of both fungi, derivatization enabled detection of 5carC in total RNA of *C. cinerea* (figure 2*a*; electronic supplementary material, figure S3A). To increase the amounts of 5carC, we treated both fungi with vitamin C, which was shown to enhance the oxidation activity of TET enzymes in mammalian cells without changing RNA levels [54,55]. Notably, the vitamin C treatment enhanced the signal in *C. cinerea*, as expected (figure 2*b*; electronic supplementary material, figure S3A), and induced the appearance of a very strong signal of the 5carC-derivative in *L. bicolor* (figure 2*d*; electronic supplementary material, figure S3A). This confirmed the presence of 5carC in *C. cinerea* and demonstrated that 5carC may appear in *L. bicolor* under certain biological circumstances.

We then investigated the distribution of 5carC among various types of RNAs: polyA tail containing RNAs, polyA-depleted large RNAs (longer than 200 nt) and small (shorter than 200 nt) RNAs. For *C. cinerea* we additionally separated large ribosomal RNAs and fractionated the pool of small RNAs into 5.8S rRNA, 5S rRNA, tRNA, a fraction of <tRNA ≥ 22 nt and RNAs shorter than 22 nt, a typical length of miRNA (electronic supplementary material, figure S3B). In *L. bicolor* treated with vitamin C, 5carC was relatively abundant in the large polyA-depleted and polyA RNAs while the signal was hardly detectable in the pool of small RNAs (shorter than 200 nt) (figure 2*d*). In untreated *C. cinerea*, the highest 5carC levels were found in small RNAs; however, the vitamin C treatment increased 5carC in the large polyA-depleted RNAs (figure 2*b*). The polyA RNAs, 5.8S rRNA and tRNA showed a weak signal after the vitamin C treatment in only one biological replicate, while 28S, 18S and 5S rRNA fractions did not contain any detectable 5carC in both conditions (figure 2*b,c*; electronic supplementary material, table S1). The 5carC levels also increased after the vitamin C treatment in the < tRNA ≥ 22 nt and shorter than 22 nt RNA fractions (figure 2*c*; electronic supplementary material, table S1). The relatively strong 5carC signal in these fractions suggests that they contribute the most to the 5carC signal in the pool of small shorter than 200 nt RNAs. Notably, we observed a 5mrC decrease and 5hmrC increase in total and small RNA after the 2 day vitamin C treatment, which potentially attests to the formation of oxi-mCs through the sequential oxidation of 5mrC by TET proteins. The 5 day vitamin C treatment further elevated the amounts of 5mrC and 5hmrC but not 5carC, perhaps as a consequence of the 5mrC reinstatement process (figure 2*b*). Interestingly, 5hmrC was not detected in the polyA RNAs of *C. cinerea* in all conditions.

Taken together, the presence of 5carC in large RNAs other than the most abundant 28S, 18S rRNAs in *C. cinerea* indicates its association with mRNAs that lack or have short polyA tails, or non-coding RNAs. Considering that polyA RNA in *L. bicolor* acquired 5carC upon vitamin C treatment, this modification might be gained under some biological conditions. It should be noted that 5carC amounts remained almost unaffected by vitamin C in the pool of small RNAs of *C. cinerea* and *L. bicolor*, demonstrating that this modification does not cause or indicate RNA degradation. Nevertheless, we cannot exclude that the weak 5carC signal in the total pool of small RNAs is the consequence of the degradation of large RNAs, or whether it comes from very short regulatory RNAs, like miRNA, which was identified in fungi (microRNA-like RNAs were found in *C. cinerea*; [56]); this remains to be elucidated.

### 3.3. Profiling of 5hmC and 5fC genome wide

**Figure 3.**
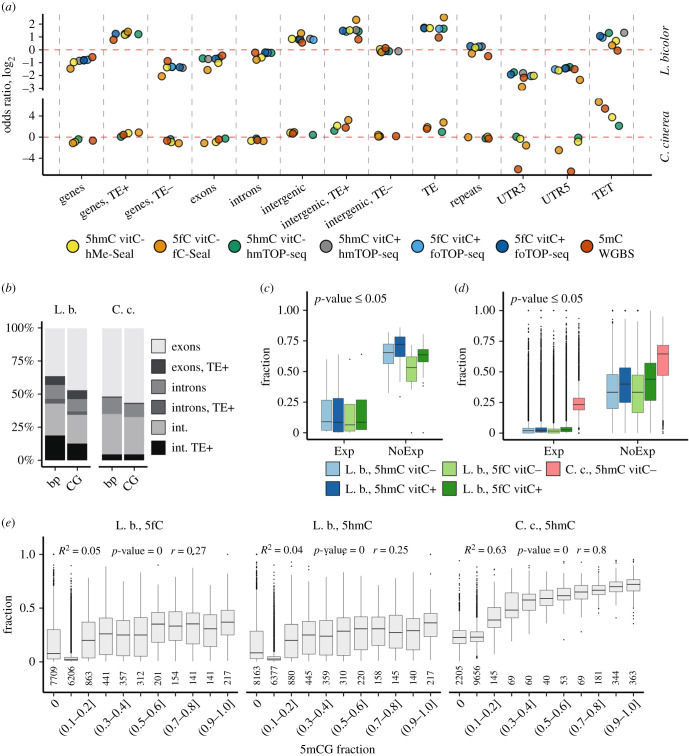
Distribution of 5hmC, 5fC and 5mC modifications in the genomes of *L. bicolor* and *C. cinerea*. (*a*) Enrichment (odds ratio (OR) log2) of the 5hmC and 5fC signal across various genomic features for hMe-Seal and fC-Seal (200-bp bins), hmTOP-seq and foTOP-seq (single CGs) and the 5mC signal from WGBS (CGs methylated more than 50% were used, [3]) data. Significance was obtained using Fisher's exact test. Non-significant estimates (*p* ≥ 0.05) are marked with an 'X'. UTR5 and UTR3, gene 5′ and 3′ untranslated regions, respectively. (*b*) Proportions (in bp and CG amounts) of genomic elements (exons, introns, intergenic regions) including and excluding TEs (TE+/TE–) in both fungi. int., intergenic areas. (*c*) Fractions of 5hmCGs and 5fCGs in expressed (TPM > 0.05, Exp) and non-expressed (NoExp) *TET* genes of *L. bicolor* and (*d*) in all genes of *L. bicolor* and *C. cinerea*. All pairwise Wilcox test *p-*values were less than or equal to the indicated *p*-value. (*e*) Fractions of oxi-mCs across gene groups stratified according to gene 5mCG fractions. Below, a number of genes within each group are presented. Only genes containing oxi-mCs were used. A linear model was used to fit the plotted data. TE, transposable elements; repeats, other repeated sequences (tandem repeats, mini and microsatellites), excluding TEs. Samples treated with vitamin C or not are marked with vitC+ or vitC–, respectively. L. b., *L. bicolor*; C. c., *C. cinerea*.

We next investigated the distribution of the most abundant oxi-mCs, 5hmC and 5fC in the genomes of *L. bicolor* and *C. cinerea* using hMe-Seal and its modified version fC-Seal, which are based on affinity enrichment of 5hmC and 5fC, respectively [9,41]. We first optimized the protocols for analysis of fungal DNA on model DNA fragments which demonstrated a good specificity for 5hmC and 5fC (Materials and methods; electronic supplementary material, figure S4). To compare genomic distributions of both oxi-mCs in *L. bicolor* and *C. cinerea*, we subjected their genomic DNA through our protocols enriching 5fC and 5hmC and the prepared libraries were sequenced at medium sequencing coverage (25× for *L. bicolor* and 45× for *C. cinerea*). In both fungi, 5hmC- and 5fC-containing genomic bins of 200 bp in length showed moderate overlap with each other (Jaccard coefficients are 0.51 and 0.33 for *L. bicolor* and *C. cinerea*, respectively). Moreover, both signals demonstrated high overlap with whole-genome bisulfite sequencing data (WGBS, [3]), which represent an aggregate 5mC and 5hmC signal (56.65% and 69.70% of the 200-bp genomic bins containing WGBS modification signal overlap with hMe-Seal, and 50.97% and 79.63% of the WGBS bins overlap with fC-Seal genomic bins for *L. bicolor* and *C. cinerea*, respectively). Enrichment analysis of 5hmC- and 5fC-containing regions with various genomic elements revealed that both modifications occurred primarily at gene-associated and intergenic TEs, and intergenic areas, similar to 5mC (figure 3*a*). This corresponds to the 5hmC profiling results in *C. cinerea* using other methods—5hmC was shown to be distributed in TEs and their surrounding regions, centromeres, non-expressed orphan and multicopy paralogous genes [29]. We also observed the enrichment of 5hmC/5fC at TET genes in both fungi that was stronger in *C. cinerea*.

As in most basidiomycetes, the most abundant TEs in both fungi are class I (retrotransposons) elements belonging to the long terminal repeat (LTR) order, of which the Gypsy and Copia families are the most numerous [30,31,57]. LTR retrotransposons are closely related in both these fungi [58]. The second most abundant TE group is represented by the terminal inverted repeat (TIR) group (class II, DNA transposons). The *L. bicolor* genome has been reported to contain larger TE families than the *C. cinerea* genome (for example, 4662 LTRs compared with 907, 511 TIRs compared with 12 reported in [59]). Furthermore, in contrast to *C. cinerea,* TEs are not restricted to subtelomeric or centromeric regions, but distribute throughout the genome forming clusters of diverse TE types [30,31]. The different genome composition of the two fungi is shown in figure 3*b*: although intergenic regions are most abundant in TEs in both fungi, a higher percentage of intergenic TEs was observed in *L. bicolor*. Of note, only a very small fraction of the annotated protein-coding genes contain TEs in *C. cinerea*, while this fraction increases up to approximately 30% in *L. bicolor*.

As genes in the fungi are relatively short (on average 1.5 kb in *L. bicolor* [31]; 1.8 kb in *C. cinerea* [30]), the medium resolution of the affinity enrichment-based methods (approx. 150–400 bp based on DNA fragment length in our experiments) may generate overlapping results of oxi-mC distribution across genomic elements, especially for TEs whose median length is 256 bp and 233 bp in *L. bicolor* and *C. cinerea*, respectively. Therefore, we next aimed to refine the distribution of 5hmC and 5fC by our single CG mapping techniques based on the TOP-seq strategy [60], putting more emphasis on *L. bicolor*, as oxi-mCs have not been analysed so far in its TE-rich genome.

To strengthen the oxi-mC signals in the fungal DNA, we performed the vitamin C treatment: HPLC-MS/MS demonstrated an increase from approximately 0.0004% to 0.0007% of 5hmC and from 0.0005% to 0.0009% of 5fC in *L. bicolor*; in *C. cinerea*, 5hmC increased from approximately 0.0002% to 0.0014% and 5fC from 0.0004% to 0.002% (electronic supplementary material, figure S5). To profile 5hmCGs in these samples, we used our recently developed hmTOP-seq approach [49]. Additionally, we developed its modified version foTOP-seq, in which the 5fC reduction to 5hmC is performed before hmTOP-seq library preparations (see electronic supplementary material, Methods), and used it for generation of 5fCG maps in *L. bicolor*. As 5mC in the *L. bicolor* and *C. cinerea* genomes occurs mainly in a CG context [3], we focused our analysis on CGs, which are distributed in all main genomic elements, with larger fractions in exons and intergenic areas (figure 3*b*). In *L. bicolor* DNA, we identified 275 922 5hmCGs and 271 761 5fCGs in two replicates of the hmTOP-seq and foTOP-seq libraries, respectively (on average 10× coverage), whose amounts increased to 315 170 5hmCGs and 349 758 5fCGs under the vitamin C treatment, as expected. In *C. cinerea* cultivated under standard conditions, we identified 663 841 5hmCGs in two replicates of the hmTOP-seq library (on average 10× coverage). The relatively high correlation between 5hmCG- and 5fCG-containing regions in various genomic elements (Pearson *r* = 0.85 for genes and 0.49 for repeats) indicated a strong overlap of 5hmC and 5fC in *L. bicolor*. The enrichment analysis of the identified oxi-mC-modified CGs across genomic elements generally coincided with the aforementioned results of hMe-Seal and fC-Seal and demonstrated similar tendencies for both vitamin C-treated and -untreated samples (figure 3*a*): 5hmCGs and 5fCGs were enriched in TEs, intergenic areas, with the most modified TEs localized in intergenic areas (electronic supplementary material, figure S6A) and TE-containing genes, and were depleted in 5′UTRs and 3′UTRs. Although exons and introns were generally oxi-mC-poor in both fungi, a slightly stronger enrichment was observed for exons in *C. cinerea*. Interestingly, although 5′UTRs and 3′UTRs in *C. cinerea* were depleted in the WGBS-acquired modification signal, they showed weak enrichment in 5hmCGs and 5fCGs. In contrast to *L. bicolor*, the genomic elements containing oxi-mCG-modified TEs comprised only a small part of all elements in *C. cinerea* (electronic supplementary material, figure S6A).

As TEs might map to many places in the fungal genomes, in parallel we analysed hm/foTOP-seq data using multi-mapping sites (see electronic supplementary material, Methods) and used them for all further analyses, except for comparisons with WGBS. In the multi-mapping approach, a much higher percentage of modified 5hmCGs/5fCGs was identified in TEs in *L. bicolor* (electronic supplementary material, figure S6A). The oxi-mCG enrichment analysis for different classes of TEs evidenced similar enrichment in retrotransposon and DNA transposon classes in both fungi; among DNA transposons, 5hmCGs/5fCGs were enriched in TIRs and in a helitron family found in *L. bicolor* in gene-rich regions [58]. For class II elements, enrichment was observed in LTR (such as Gypsy, Copia) and some non-LTR families (DIRS or LINE) (electronic supplementary material, figure S6B). Although the abundance of TE families is very different, with LTRs being the most numerous, comparable enrichment of all families suggests potentially similar regulation of TEs through oxi-mCs.

To compare the distribution of oxi-mCGs and their predecessor 5mCGs across genes and TEs, we used our strategy to calculate modified CG fractions, as described previously [61,62]. Importantly, for WGBS data, the calculated 5mCG fractions across genes correlated well with 5mCG methylation values (0.94 for *C. cinerea* and 0.54 for *L. bicolor*). Therefore, this strategy enabled the direct comparison of different data and modifications measured by WGBS and our semi-quantitative TOP-seq-based methods. The vast majority of genes in *C. cinerea* contained 5hmCGs (13 185 single-mapping and 13 215 multi-mapping genes of the analysed 13 237 genes), while of 22 910 analysed genes in *L. bicolor* oxi-mCs were found in 19 760 and 20 320 genes using a single-mapping and multi-mapping approach, respectively. We observed a good coincidence between oxi-mCGs and 5mCGs that was stronger in *C. cinerea* (figure 3*e*). In *C. cinerea,* 5mCG-containing genes distributed bimodally: a large group of genes (approx. 11 900) showed the absence or low 5mCG (0–0.1) and average 0.2 5hmCG fractions, and the second most abundant group consisted of highly 5mCG- and 5hmCG-enriched genes (approx. 890 genes with ≥ 0.7 5mCG and an average of 0.7 5hmCG fractions). In *L. bicolor*, the majority of genes (approx. 19 500) demonstrated low 5mCG and oxi-mCG fractions (0–0.1 5mCG and an average of 0.09 oxi-mCG), and the genes with medium 5mCG and oxi-mCG fractions (greater than 0.2 but less than 0.7 5mCG and an average of 0.23 oxi-mCG) outnumbered the highly modified gene group (greater than 0.7 5mCG and an average of 0.26 oxi-mCG fractions). It should be noted that a much larger group of unmethylated but oxi-mCG-containing genes was observed in *L. bicolor* than in *C. cinerea* (7709 5hmCG- and 8163 5fCG-containing genes in *L. bicolor* and 2205 5hmCG genes in *C. cinerea*), suggesting a distinct turnover of 5mC oxidation products in the two fungi (figure 3*e*). Although 14 396 of 19 760 oxi-mC-containing genes in *L. bicolor* possess both 5hmC and 5fC modifications, the genes modified with a single type of oxi-mCGs were also detected (3018 and 2346 genes with 5hmCGs and 5fCGs, respectively).

InterProScan analysis provided GO terms to only approximately 10% of the 10% most oxi-mC-modified genes of *L. bicolor*, and showed links to many different cellular processes (more than 40 GO terms), indicating that most likely 5hmCGs/5fCGs are not associated with particular genes. More genes (10–17) were found in protein dimerization activity (GO:0046983), nucleic acid binding (GO:0003676), the G protein-coupled receptor signalling pathway (GO:0007186), GTPase activity (GO:0003924), guanyl nucleotide binding (GO:0019001), G-protein beta/gamma-subunit complex binding (GO:0031683), ATP binding (GO:0005524) and catalysis of the hydrolysis of ester linkages within nucleic acids (GO:0004518) functions and processes. Of genes not defined by GO terms, but with an identified domain or motif, the majority were characterized as transposases, also belonging to the oxygenase domain of the 2OGFeDO superfamily (based on Pfam annotations).

### 3.4. Association of 5hmC/5fC with gene expression

**Figure 4.**
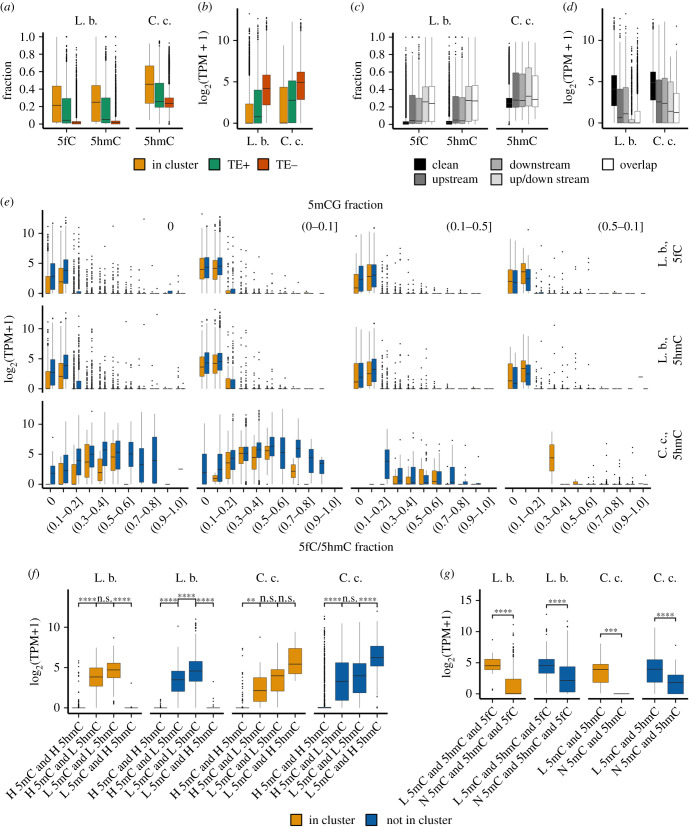
Relationship of 5hmC, 5fC and 5mC with gene expression in *L. bicolor* and *C. cinerea*. (*a*) 5hmCG and 5fCG fractions and (*b*) expression of all genes which are associated with TEs (TE+, distance less than 1 kb) or not (TE–), or present in TE clusters (In cluster). Kruskal–Wallis and one-way ANOVA (for fractions and expression, respectively) were used to test differences within groups (*p*-value ≤ 0.05). (*c*) 5hmCG and 5fCG fractions and (*d*) expression of genes grouped according to the position of TEs (clean, no TE detected within a 1 kb distance; upstream or downstream, TE detected within 1 kb upstream or downstream from a gene; up/downstream, TE detected upstream and downstream from a gene; overlap, TE overlaps a gene). Kruskal–Wallis and one-way ANOVA (for fractions and expression, respectively) were used to test differences within groups (*p*-value ≤ 0.05). (*e*) Expression (log2(TPM+1)) stratified according to 5fCG and 5hmCG gene fractions in differentially methylated genes (5mCG fractions from WGBS data shown above the graphs). (*f*) Comparison of the expression of differently modified genes grouped according to high (H) and low (L) gene 5mCG and 5hmCG fractions in TE clusters and outside them. Asterisks indicate *p*-values calculated using the *t*-test. (*g*) Expression of genes containing low levels (L) of all three 5mC-, 5fC- and 5hmC-modified CGs in *L. bicolor* and low 5mCGs/5hmCGs in *C. cinerea*, and without (N) these modifications inside and outside of TE clusters. Asterisks indicate *p*-values calculated using the *t*-test. L. b., *L. bicolor*; C. c., *C. cinerea*; n.s., *p*-value > 0.05; *p < 0.05; **p ≤ 0.01; ***p ≤ 0.001; ****p ≤ 0.0001.

To evaluate the impact of oxi-mCs on gene expression in fungi, we performed whole-transcriptome sequencing of *L. bicolor* mycelium cultivated under normal and elevated vitamin C conditions. For *C. cinerea*, we used the publicly available polyA RNA-seq data of the mycelium cultivated under standard conditions [43]. Although most of the *L. bicolor TET* genes identified in our analysis (46 genes, see Methods) possess 5fC/5hmC modifications (figure 3*c*), only four of them with low oxi-mCG fractions showed detectable expression (TPM > 0.05) and could potentially be involved in the production of oxi-mCs (figure 3*c*). Generally, we determined the preferential association of oxi-mCs with non-expressed genes (figure 3*d*). Moreover, all multi-copy genes were more oxi-mC-modified and less expressed than single-copy genes (electronic supplementary material, figure S6C and D), which is consistent with the reported results of 5hmC in *C. cinerea* [29]. As TE-containing genes and intergenic regions were most enriched in 5hmCGs/5fCGs (figure 3*a*), we further explored the expression and modification of genes depending on the presence of TEs. It is known that insertion of TEs or even proximity to them downregulates genes in fungi [63]. Although TEs in fungi are globally transcriptionally inactivated by DNA methylation [64] and heterochromatin formation [65,66], the impact of 5mC oxidation on the activity of TEs is unknown. We found that genes and their upstream regions, which might contain promoters, were more 5hmCG/5fCG-modified when they overlapped TEs in both fungi (figure 4*a*; electronic supplementary material, figure S8A), and, consequently, such genes were less expressed (figure 4*b*). *Laccaria bicolor* genes that overlapped TEs or were surrounded by them demonstrated the highest oxi-mC modification and the lowest expression levels (figure 4*c,d*). As TEs contain relatively high fractions of oxi-mCGs (electronic supplementary material, figure S7A), this strongly confirms the TE influence on gene 5fC/5hmC modification levels and, concomitantly, the negative impact on expression.

It has been reported that more methylated, less active genes distribute in TE clusters in some fungi [63,64]. TE clusters were detected in the *L. bicolor* genome [34]. For comparative analysis of *L. bicolor* and *C. cinerea*, we *de novo* identified TE clusters, which we defined as regions of at least 5 kb in length with repeat content of at least 50% (see electronic supplementary material, Methods). As expected, a considerably higher number of clusters were found in *L. bicolor*: 207 and 82 TE clusters in *L. bicolor* and *C. cinerea,* respectively, covering 20 966 119 and 2 578 862 bp (35.48% and 7.2% of the genome) and having on average 60 and eight TEs per cluster. We observed that TEs in clusters demonstrated higher 5hmCG/5fCG fractions than solitary TEs (electronic supplementary material, figure S7A), and highly oxi-mCG-modified TEs associated with low expression (electronic supplementary material, figure S7B). In accordance with high oxi-mCG fractions of TEs in clusters, genes localized in TE clusters (and their upstream regions) were most enriched in 5fCG/5hmCGs (figure 4*a* and electronic supplementary material, figure S8A) and least expressed in relation to genes outside of clusters or without TEs (figure 4*b*; electronic supplementary material, figures S7C, S8B, S8C).

Generally, the majority of TE cluster-associated genes in *L. bicolor* (4312 out of 7095) were found to be non-expressed (average 0.28 5hmCG/5fCG and 0.11 5mCG fractions), while only 3618 out of 15 815 genes outside of TE clusters were silenced (average 0.24 5hmCG/5fCG and 0.13 5mCG fractions), which attests to the silencing effect of TEs. The measured fractions of modified cytosines of expressed genes in clusters were slightly higher than those outside them and consisted of on average 0.06 5hmCG, 0.06 5fCG and 0.07 5mCG (0.03 5hmCG, 0.02 5fCG and 0.04 5mCG outside of clusters).

As genes and TEs included all three cytosine states, 5mC, 5hmC, 5fC, we further sought to investigate the interplay among individual cytosine modifications in the regulation of gene expression. We grouped all genes according to 5mCG fractions and evaluated their expression as a function of increasing oxi-mC fractions. Strikingly, the relationship between 5hmCG amounts and expression differed in *L. bicolor* and *C. cinerea* (figure 4*e*). A positive relationship was detected for lowly methylated genes (5mCG fraction 0–0.1) in *C. cinerea,* in line with the observed positive influence of 5hmC on the expression in mammals [67], while expression was weak or absent in highly methylated and hydroxymethylated genes (fractions > 0.5). In *L. bicolor*, the positive association between expression and 5hmCG/5fCG fractions was only noticeable when comparing the two gene groups—without oxi-mCGs and with low oxi-mCG fractions (up to 0.1), whereas genes with higher than 0.1 5hmCG/5fCG fractions were mostly silenced irrespective of their 5mCG content. The same trend was observed for genes associated with TE clusters and outside them, except that expression levels were lower inside clusters. To assess the relevance of the CG-fraction measure for gene modification evaluations, we additionally calculated CG-coverage values across the same 5mCG groups: both sets of data generally corresponded, except for the groups with higher 5hmCG modification values, which showed weaker downregulation of lowly methylated genes in *L. bicolor* and did not show strong downregulation in *C. cinerea* (electronic supplementary material, figure S7D). This suggests the presence of genes that contain a low number of highly modified, i.e. highly covered, 5hmCG sites and indicates that complementary information could be collected using the two parameters of TOP-seq data analysis.

The close-up view at genes with high or low fractions of different cytosine modifications in *L. bicolor* and *C. cinerea* again revealed that, in *L. bicolor*, both oxidized 5-methylcytosine forms (data shown only for 5hmC) strongly suppress genes in TE clusters and outside them: for both highly and lowly methylated gene groups, only the genes possessing a low 5hmCG fraction showed expression while highly 5hmCG-modified genes were silenced (figure 4*f*). By contrast, higher 5hmCG levels mark more active genes when their methylation level is low (*p-*value < 0.01) in *C. cinerea*, but abundant 5hmCGs and 5mCGs act synergically in repression of genes (at low gene 5hmCG levels, higher 5mCG amounts tend to downregulate genes as compared with low 5mCG levels), with a stronger repression effect of high 5hmCG fractions (*p-*value < 0.01). On the other hand, despite the observed silencing role of oxi-mCs in both fungi and 5mC in *C. cinerea*, genes containing low levels of all cytosine modifications were found to be more intensively expressed than genes without modifications (*p-*values < 0.001, figure 4*g*). The observed differences between the fungi might suggest the presence of different mechanisms for regulation of more abundant TEs in *L. bicolor* than in *C. cinerea*. However, we cannot exclude that the differences arise as a result of the poor annotation of genes and TEs in the *L. bicolor* genome [31,63].

## 4. Discussion

To understand the outcomes that might arise from the presence of many copies of *TET-JPB* genes in *L. bicolor* and *C. cinerea*, we investigated the distribution of the 5mC oxidative forms in the nucleic acids of the fungal mycelium. We detected all three oxi-mCs in DNA of both fungi, with similar levels of 5hmC and 5fC, which is in contrast to much higher 5hmC abundance in mammalian DNA as compared with 5fC (figure 1*d*). This might indicate different turnover or biological roles of 5hmC and 5fC in the fungi. Contrary to mammals, *L. bicolor* and *C. cinerea* have many *TET-JPB* genes (47–74 genes) in comparison with only three genes in mammals [28,67]. Despite numerous *TET-JBP* genes, only a few of them showed weak expression (this study and [29]); thus, the observed relatively low levels of oxi-mCs in the genomes of *L. bicolor* and *C. cinerea* are not unexpected. The amount of 5caC in DNA of *C. cinerea* was the lowest among the three oxi-mC modifications (figure 1*b,d*), in contrast to the most abundant 5caC reported by [29]. The detected discrepancy of 5caC proportions in HPLC-MS/MS and western dot–blot analyses might be explained by a cross-reactivity of the used 5caC antibodies, questioning the reliability of antibody-based methods for analysis of fungal samples. For this reason, we focused on the comprehensive characterization of oxi-mCs in the DNA and RNA of *L. bicolor* and *C. cinerea* by HPLC-MS/MS.

We discovered 5carC in *C. cinerea* RNA and developed a chemical derivatization method to facilitate its detection by HPLC-MS/MS—the rarest and the least investigated oxi-mC. Among different RNA types, 5carC was observed in polyA-depleted large RNAs, pointing to its potential association with mRNAs that lack or have short polyA tails or non-coding RNAs. It has been shown that some fungi contain a significant fraction of mRNAs lacking polyA tails [68], and short polyA tails are common in highly expressed genes across eukaryotes [69]. An apparent 5carC signal was also identified in *C. cinerea* small RNAs, with the largest 5carC amounts found in RNAs shorter than tRNA. The treatment of the fungal mycelium with vitamin C induced an increase of oxi-mCs in its RNA and DNA, supporting the potential involvement of TET proteins in the 5mC oxidation. Under standard cultivation conditions, 5carC was not detected in *L. bicolor* by our method*,* but the vitamin C treatment facilitated its substantial increase in large and polyA RNAs, indicating that 5carC can be formed at higher cellular concentrations of vitamin C (fungi naturally synthesize vitamin C or its analogues [70]), or, for example, following interaction with a plant. Surprisingly, our data showed the absence of 5hmrC in the polyA RNA fraction of *C. cinerea*, which was detected following the vitamin C treatment only in *L. bicolor*, suggesting that oxi-mCs play different roles in fungi than in mammals, where the highest percentage of 5carC and 5hmrC was estimated in the polyA RNAs [6]. Although the levels of 5carC in total RNA of *C. cinerea* (approx. 0.00001–0.00005% of G) and *L. bicolor* (approx. 0.0004%) are low, they fall within a similar range to those determined in different mammalian tissues (0.00004–0.0005%; [6]) (figure 1*d*). The relatively weak TET activity towards 5caC generation, as reported by *in vitro* analysis of *C. cinerea* TET proteins on DNA [71], might be responsible for the lowest amounts of 5carC among all oxi-mCs, or 5caC might be actively removed from DNA and RNA by excision mechanisms, such as thymine DNA glycosylases or direct decarboxylation to cytosine [72]. However, to date, the activity of thymine DNA glycosylases (a potential gene was predicted in *C. cinerea*; [29]) or other excision mechanisms have not yet been discovered in fungi.

Our investigation of the presence of 5carC in fungal RNA appends valuable information about this poorly explored modification, though the mechanisms of its biological functioning are yet to be determined. 5carC might influence RNA geometry, similarly to 5caC in DNA, by widening the minor groove and loosening its double-stranded parts as opposed to more stabilizing 5mC [73]. Other cytosine modifications in RNA are shown to affect RNA properties, stability and functions in various organisms. 5mrC in mRNAs enables their transport from the nucleus in human HeLa cells, or to distant body parts of the plant *Arabidopsis thaliana* [74]. Some studies predicted the 5mrC influence on mRNA stability [74]. In addition, 5mrC was found to affect the binding of regulatory long non-coding RNAs to chromatin-modifying complexes [75]. In contrast to 5mrC, which decreases translation of mRNA, 5hmrC can favour it in *Drosophila* [23]. Interestingly, 5hmrC was shown to destabilize murine endogenous retrovirus transcripts [75,76].

TEs comprise a larger part of the genome in ectomycorrhizal *L. bicolor* (up to 37.9% [63]), compared with saprotrophic *C. cinerea* (approx. 6%, [30]). The expansion of TEs in fungi is thought to be related to mutualistic or parasitic interactions with plants, thereby contributing to genetic variation that is beneficial for adaptation to a host plant [57], and significantly higher TE content in ectomycorrhizal symbionts was found compared with other fungal lifestyles [77]. Our hmTOP-seq-based [49] profiling of 5hmC- and 5fC-modified CG dinucleotides in DNA of both fungi showed their accumulation at TEs, which is in accordance with the published distribution of 5mC [3] and 5hmC in *C. cinerea* [29]. We found that the majority of genes in the fungi possess oxi-mCs. The association of genes with oxi-mC-rich TEs causes their suppression and affects gene 5hmC/5fC modification levels. The performed separate analysis for the genes localized inside and outside of TE clusters demonstrated the highest 5hmC/5fC modification levels in clusters, especially in non-expressed genes, which is in line with 5mC analysis of other fungi [63,64]. By contrast, *C. cinerea* genes displayed gradually increasing expression with higher 5hmC modification levels irrespective of the TE presence.

Importantly, using our strategy to analyse the fractions of modified CGs, we were able to directly compare the amounts of the three cytosine states—5mC, 5hmC and 5fC—and their relationship with gene expression regulation. The amounts of all three are positively correlated in *L. bicolor*—this is in direct contrast to the oxi-mC proportions in mammals, where the inverse correlation was estimated between 5fC and 5hmC, or between 5hmC/5fC and 5mC levels [17,78]. Strikingly, the analysis of individual cytosine modifications across genes revealed that high gene 5hmC or 5fC but not 5mC modification exerts a strong suppressive effect in *L. bicolor*. By contrast, more active genes are marked by higher 5hmC and low 5mC levels in *C. cinerea*, and only high 5mCG levels start to act negatively on expression along with high gene 5hmCG levels. Despite the strong repressive role of oxi-mCGs observed in both fungi, low levels of 5mC/5hmC/5fC modification are required to induce expression.

To our knowledge, this is the first detailed analysis of the relationship between 5mC and its oxidized derivatives 5hmC/5fC and expression in fungi. The observed silencing of genes whose high oxi-mC levels are caused by highly 5hmCG/5fCG-modified TEs suggests the oxidation of 5mC as an additional regulation/suppression mechanism that fungi might accomplish, for example by recruiting repressive proteins to TEs, as has been shown for TET proteins in mammals [79]. This mechanism could also be important for autoregulation of the expanded pool of TE-associated [26,27] and 5hmC/5fC-modified *TET-JPB* genes. Diverse impacts of oxi-mCs and 5mC on expression in *L. bicolor* and *C. cinerea* acknowledge the variety of roles that cytosine modification might play in different species of fungi [3,80]. Several mechanisms could have arisen in *L. bicolor* during its evolution in symbiosis with plants, which, together with the accumulation of TEs contributing to adaptation, activate the strong control over deleterious effects of abundant TEs.

The established distribution of the 5mC and oxi-mC modifications along with gene expression profiles of the two fungi provide a unique resource to study epigenetic mechanisms and highlight the importance of individual oxidative cytosine modifications. Further research is needed to gain deeper insights into the diversity of roles and distribution of oxi-mCs in other developmental stages of *L. bicolor* and *C. cinerea* and in many different fungi.

## Data Availability

Raw and processed sequencing data reported in this paper are available online at GEO: GSE185551. Figures and data analysis source codes and data used to produce final figures presented in this manuscript are available on Zenodo at https://doi.org/10.5281/zenodo.5846594. The data are provided in the electronic supplementary material [81].
